# An Electrochemical DNA Biosensor for Carcinogenicity of Anticancer Compounds Based on Competition between Methylene Blue and Oligonucleotides

**DOI:** 10.3390/s19235111

**Published:** 2019-11-22

**Authors:** Nor Diyana Md. Sani, Eda Yuhana Ariffin, Wong Sheryn, Mohd Asyraf Shamsuddin, Lee Yook Heng, Jalifah Latip, Siti Aishah Hasbullah, Nurul Izzaty Hassan

**Affiliations:** 1 Sanichem Resources Sdn Bhd, No 7 & 7a, Jalan Timur 6/1a, Mercato Enstek, Bandar Enstek NSN 71060, Malaysia; diyana@sanichem.com.my; 2 Center for Advanced Materials & Renewable Resources, Faculty of Science & Technology, Universiti Kebangsaan Malaysia, Bandar Baru Bangi 43600, Selangor Darul Ehsan, Malaysia; edayuhana@ukm.edu.my (E.Y.A.); w.sheryn@gmail.com (W.S.); asyrafsham@yahoo.com.my (M.A.S.); yhl1000@ukm.edu.my (L.Y.H.); jalifah@ukm.edu.my (J.L.); aishah80@ukm.edu.my (S.A.H.)

**Keywords:** toxicity, electrochemical, DNA biosensor, guanine, carcinogen

## Abstract

A toxicity electrochemical DNA biosensor has been constructed for the detection of carcinogens using 24 base guanine DNA rich single stranded DNA, and methylene blue (MB) as the electroactive indicator. This amine terminated ssDNA was immobilized onto silica nanospheres and deposited on gold nanoparticle modified carbon-paste screen printed electrodes (SPEs). The modified SPE was initially exposed to a carcinogen, followed by immersion in methylene blue for an optimized duration. The biosensor response was measured using differential pulse voltammetry. The performance of the biosensor was identified on several anti-cancer compounds. The toxicity DNA biosensor demonstrated a linear response range to the cadmium chloride from 0.0005 ppm to 0.01 ppm (R^2^ = 0.928) with a limit of detection at 0.0004 ppm. The biosensor also exhibited its versatility to screen the carcinogenicity of potential anti-cancer compounds.

## 1. Introduction

Chemicals that possess carcinogenicity are classified by various bodies including the International Agency for Research on Cancer (IARC). Carcinogens are chemicals that have the potential to cause cancer. This is due to their ability to bind onto the DNA and mutate it. Due to this tendency, the binding property can be used as the basis for the construction of a toxicity biosensor. In general, there are two major problems encountered when constructing a toxicity DNA biosensor, which are: finding a suitable type of DNA and finding an ideal immobilization matrix for the particular DNA.

There have been many types of DNAs utilized by researchers, and two frequently used DNAs are calf-thymus DNA [1] and salmon sperm DNA [2]. These DNAs are usually favored since they can be extracted in large quantities, are able to replace human DNA in many applications, and are commercially accessible. Nevertheless, these DNAs have very long DNA sequences, which is not ideal in building a DNA biosensor as it may fold onto itself when immobilized onto an electrode [3]. This will reduce the DNA surface that is able to interact with the carcinogen. In addition, molecules with low molecular weights are most likely to display the tendency to bind to short DNA sequences of 10 to 16 bases long [4]. On the other hand, DNA with abounding guanine bases and repeating base sequences preferred minor groove binding [5] over intercalation with carcinogens. Meanwhile, the utilization of single stranded DNAs (ssDNA) in the construction of biosensors yielded stronger nucleobases responses [6] compared to double stranded DNAs (dsDNA). This is because ssDNA has bases that are exposed to the environment which enables easy access for carcinogen interaction. The Del Carlo group has used ssDNA taken from salmon sperm to detect the presence of benzo(a)pyrene oxidation products [7]. A screen-printed graphite electrode was pretreated in a potential of +1.6 V for 120 s and then at +1.8 V for 60 s, before being immersed in a DNA solution at +0.5 V for 120 s. This biosensor successfully achieved a dynamic range of 0.01 µg/mL to 0.04 µg/mL. Hianik and co-workers focused on the immobilization of aptamer and poly(neutral red) and carboxylated pillar[5]arene for sensitive carcinogenic effect determination of aflatoxins M1 in milk and milk products from 5 to 120 ng/mL with the limit of detection of 0.5–1 ng/mL [8].

Thus, choosing a suitable matrix and immobilization method is a pivotal element in the fabrication of a DNA biosensor [9], as they assure the biomolecules remain stable on the matrix or electrode surface to establish a reaction with the intended analyte. In the past, various types of materials such as carbon nanotubes, conductive polymers, metal nanoparticles, and composites from electroactive materials [10] have been considered to modify electrodes such as gold (Au), silver (Ag), platinum (Pt), glassy carbon, and indium tin oxide (ITO). 

When a suitable matrix is used, the developed biosensor will possess high sensitivity in addition to excellent selectivity and stability. The immobilization of DNA onto a matrix requires strong binding between the matrix and DNA, without damaging the properties of the DNA biomolecule [11]. There are many methods frequently used to immobilize DNA such as electrochemical and physical adsorption, electrochemical entrapment, and covalent bonding [7]. Nonetheless, adsorption and entrapment methods produce weak molecular binding between the biomolecule and immobilization matrix. This weak interaction results in leaching of the immobilized biomolecule, thus decreasing the life span or shelf life and stability of the biosensor [12]. Contrarily, the covalent bonding method is known to form stronger binding and more preferred for the development of high performance DNA biosensors [13].

The application of nanoparticles and microspheres as immobilization matrices will contribute to the enhancement of a biosensor’s performance more than membrane like immobilization matrices. This is due to the three-dimensional structure that provides a large surface area for biomolecule immobilization as compared to two-dimensional membranous structures [14]. The unique property of silica nanospheres has been long exploited to immobilize enzymes and DNA biomolecules [15,16]. The uniform size distribution of silica nanospheres promoted homogeneity of the immobilized reagent molecules on the nanosphere’s surface, thereby enhancing the sensing response [17]. 

Nitrogen-containing heterocycles such as pyridine, pyrazole, quinolone, and indoles are essential building blocks to numerous bioactive compounds. These compounds have shown remarkable anti-bacterial and anti-fungal activities against distinct bacteria and fungi [18,19,20]. In addition to that, these derivatives also possess remarkable antitumor activities and may be useful in the treatment of numerous cancers [21,22,23,24,25]. However, compounds with potential as antitumor agents are only desirable for chemotherapeutic use with reduced toxicity [26]. Therefore, it is important to test the carcinogenicity of these compounds. 

In this work, silica nanospheres (SNs) were chosen as the immobilization matrix of DNA in this study by covalently linking amine DNA to aminated silica nanospheres (SNs) via a glutaraldehyde bifunctional cross-linker. The biosensor used a special 24 base guanine rich DNA and methylene blue (MB) as its redox indicator. This sequence was designed based on the p53 tumor suppressor gene where the sequence CGG comes up many times. It is due to the repeating guanine bases that this gene becomes a target for mutation [27]. The biosensor is employed as a complementary toxicity assessment on carcinogenicity of fourteen biological potential *N*-heterocyclic compounds that were previously synthesized in the group. 

## 2. Materials and Methods

### 2.1. Instrumentation

All differential pulse voltammetry (DPV) measurements were conducted using an Autolab PGSTAT and GPES software package. The measurements were conducted in 0.15 M potassium phosphate buffer solution (PBS) at pH 7. The electrochemical system consists of a screen-printed electrode (SPE), carbon pencil electrode, and Ag/AgCl as reference electrode. The SPEs were scanned from −0.4 V to 0 V with step potential of 0.01 V and scan rate of 0.005 V/s.

### 2.2. Chemicals

The reagents 2,3-aminopropyl-triethoxysilane (APTS, 99%), glutaraldehyde, DNA, gold colloid (AuNPs, 20 nm diameter), potassium chloride (KCl), sulfuric acid (H_2_SO_4_), and carcinogen compounds (formaldehyde and acrylamide) were purchased from Sigma Aldrich (Saint Louis, MO, USA). Methylene blue (MB), ammonia (25%), and ethanol (95%) were purchased from Systerm (Selangor, Malaysia) whereas tetraethyl orthosilicate (TEOS, 98%) was purchased from Fluka (Leicestershire, UK). Both potassium dihydrogen phosphate (KH_2_PO_4_) and potassium ferricyanide (K_3_[Fe(CN)_6_]) were obtained from Merck (Darmstadt, Germany). In addition, dipotassium hydrogen phosphate (K_2_HPO_4_) was obtained from BDH Laboratory Supplies (Poole, England). All standard buffer and chemical solutions were prepared freshly using MilliQ-deionized water.

### 2.3. Preparation of Solutions

MB solution (1 mM) was prepared by dissolving powdered MB in deionized water. In order to prepare DNA solutions of various concentrations, DNA stock solution (100 μM) was diluted with potassium phosphate buffer (PBS), pH 7.0 and stored at −5 °C when not in use. The DNA sequence used in this study is 24 bases of guanine rich oligonucleotide with the full sequence of CGG-CGG-CGG-CGG-CGG-CGG-CGG-CGG.

### 2.4. Synthesis of Aminated SNs

A mixture of deionized water (2 mL), ammonium solution (5 mL) and ethanol (20 mL) were sonicated for 10 min followed by the addition of TEOS (2 mL) and ethanol (20 mL) and sonicated for another 40 min at 55 °C. The mixture was treated with 2 mL of APTS (99%) with overnight stirring. Next, the aminated SNs solution was sequentially washed with ethanol and deionized water by centrifugation of 4000 rpm for 20 min each. The aminated SNs slurry were collected and air-dried for 24 h.

### 2.5. Fabrication of DNA Biosensor

**Figure 1:**
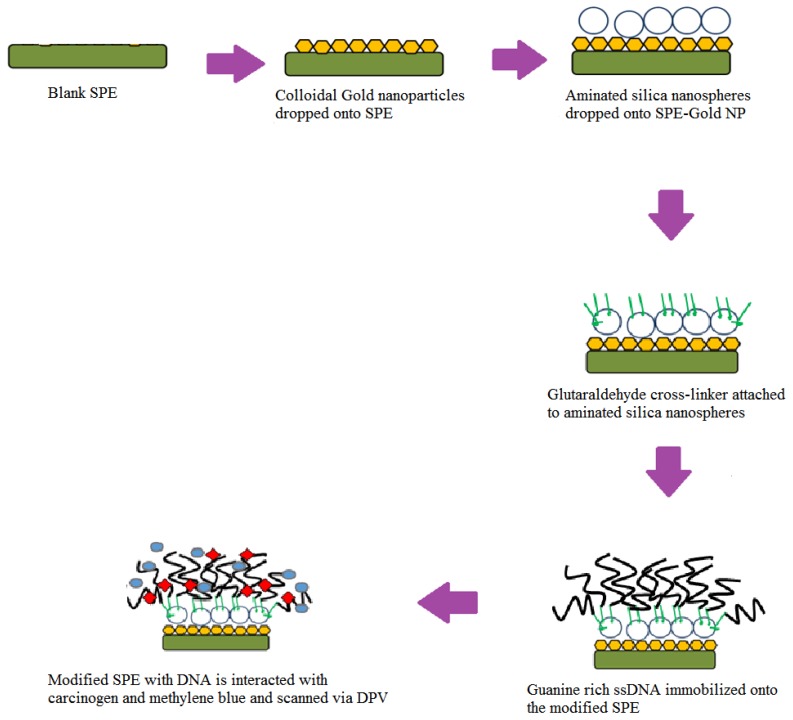
The fabrication process of the DNA biosensor starting from blank screen-printed electrode (SPE) to the immobilization of DNA onto the modified (SPE-Gold nanoparticles) before being exposed with carcinogen and methylene blue (MB). Differential pulse voltammetry (DPV).

We dropped 15 µL of stock colloidal AuNPs (0.0167 µg/mL) onto a carbon based screen-printed electrode (SPE) and dried overnight. Next, 10 µL from 1.5 mg of aminated SNs that were suspended in 1 mL ethanol (98%) were dropped onto the SPE and dried under ambient conditions, followed by immersion in 10% glutaraldehyde for 1 h. The modified SPE was then immersed in 400 µL of guanine rich DNA (10 µM) overnight to ensure the amine-terminated DNA bonded covalently to aminated SNs via a glutaraldehyde linker. The modified SPE was then immersed in a carcinogen solution, followed by immersion in MB indicator. Finally, the SPE was washed to remove any unbound MB and DNA molecules before being scanned using DPV from −0.4 V to 0 V with a scan rate of 0.005 V/s in 0.05 M PBS (pH 7). Figure 1 illustrates the stepwise process to fabricate the DNA biosensor.

### 2.6. Performance of DNA Biosensor with Synthesized Bioactive Compounds

**Figure 2:**
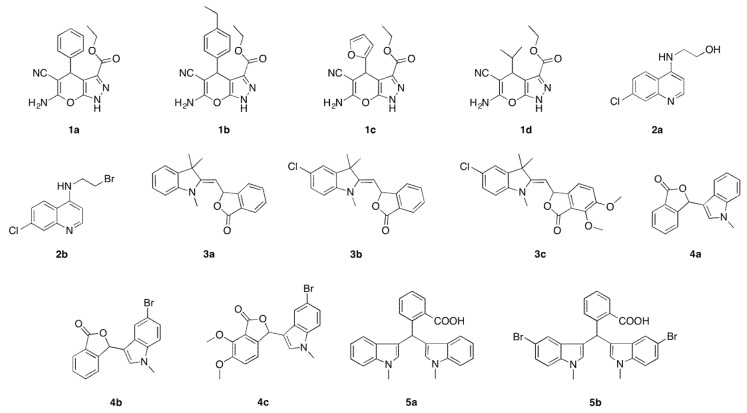
Structure of *N*-heterocyclic compounds comprising pyranopyrazole (**1a**–**d**), quinoline (**2a**–**b**), phthalide and bisindole (**3a**–**5b**) moieties used for carcinogenic detection.

*N*-heterocyclic compounds that were synthesized in our laboratory are shown in Figure 2. Compounds **1a**–**d** were synthesized following a procedure described in the literature [28] using diethyl oxaloacetate, hydrazine, aldehyde, and malanonitrile, whereas the preparation of quinoline derivatives (**2a**) have been reported by Bhagat and co-workers [29]. The insertion of a bromine group can be achieved in the presence of hydrogen bromide under a catalytic system to furnish compound **2b** following the existing methodology by the group of Aminake [30]. Meanwhile, we recently reported the preparation of phthalide and bisindoles derivatives **3a**–**c**, **4a**–**c**, and **5b** [31,32]. These compounds were tested with an electrochemical toxicity DNA biosensor using 24 base guanine-rich: CGG-CGG-CGG-CGG-CGG-CGG-CGG-CGG and methylene blue as a toxicity indicator at various concentrations from 0.0005–0.1 ppm.

## 3. Results and Discussions

**Figure 3:**
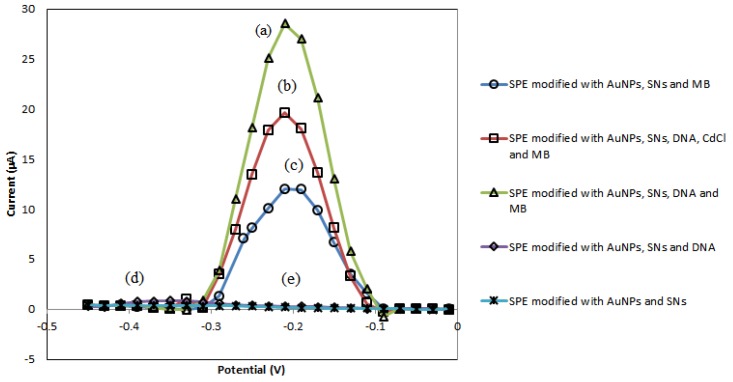
(**a**) SPE modified with AuNPs, silica nanospheres (SNs), DNA and MB. (**b**) SPE modified with AuNPs, SNs, DNA, cadmium chloride (CdCl) and MB. (**c**) SPE modified with AuNPs, SNs and MB. (**d**) SPE modified with AuNPs, SNs and DNA. (**e**) SPE modified with AuNPs and SNs.

The peak signals of DPV that are observed at −0.25 V potential in Figure 3 represents the potential of MB oxidation. The highest peak was achieved in the presence of DNA as it enabled more MB molecules to bind to the modified SPE (Figure 3a). However, there is a decrease in the peak height (Figure 3b) when carcinogen compound was added because the carcinogen analyte competes with the MB molecules to bind to the DNA. This causes less MB molecules to bind to the DNA, hence giving a smaller peak signal. Furthermore, both Figure 3a,b show that the amine terminated DNA was successfully bonded covalently to the aminated SNs via glutaraldehyde linker. Figure 3c–e act as control. On the other hand, there is no peak present when MB was not added on the modified SPE. 

The MB toxicity indicator preferably binds to the guanine bases and phosphate backbone of DNA through electrostatic forces [33]. This phenomenon was observable using the UV-visible spectrophotometer, where there was quenching of MB signals towards the guanine bases. The signals obtained when MB was interacted with repeated adenine, cytosine and thymine bases DNA sequences, respectively were lower compared to repeated guanine nucleotides. 

### 3.1. Effect of the Amount of AuNPs, SNs, and Glutaraldehyde Linkers

**Figure 4:**
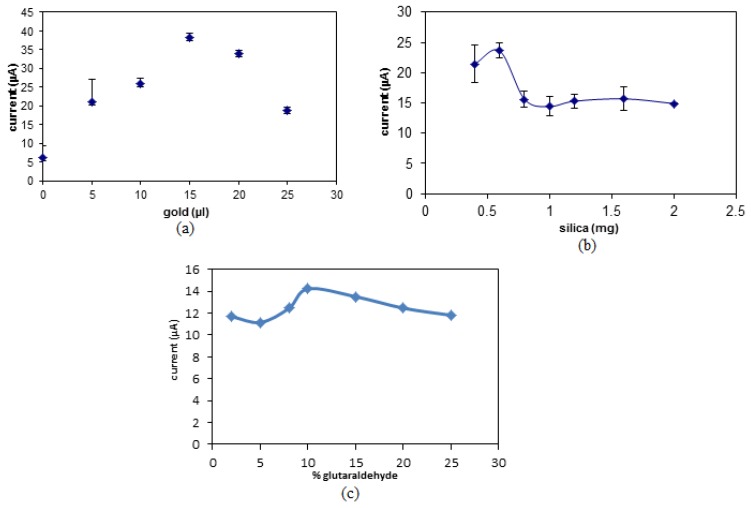
The optimization of the amount of (**a**) AuNPs, (**b**) SNs, and (**c**) glutaraldehyde linkers to be used for the biosensor.

The SNs used in this study are non-conductive, hence, they does not allow any charge transfer between the working electrode and redox indicator [15]. In order to overcome the prior issue, AuNPs were used as a signal enhancer due to their ability to transfer charges effectively. Therefore, it is important to optimize the amount of AuNPs in order to enhance the performance of the biosensor. The presence of AuNPs will increase the number of conduction pathways, which will enable more charge and hence the number of electrons to be transferred thus amplifying the biosensor’s signal [34]. This will also increase the biosensor’s sensitivity and detection limit [35]. Figure 4a shows the effect of different volumes of AuNPs (0.0167 µg/mL) towards the DPV peak signal. It can be observed that the peak signal increases up to 15 µL of AuNPs and starts to plateau. This shows that the optimum amount of AuNPs to be used in the biosensor is 15 µL, which is equivalent to 0.00025 µg/mL (Figure 4a). 

Aminated SNs were used in this study as the immobilization matrix for amine-terminated DNA via glutaraldehyde linkers. The DNA binds to the matrix through covalent bonding and the spherical shape of SNs increases the surface area to allow higher binding of the DNA molecules [36]. In Figure 4b, it can be observed that there is an increase in the DPV peak signal with increasing amounts of SNs from 0.4 mg to 0.6 mg. Beyond that, the DPV peak signal decreases and becomes constant. The increase in the biosensor response was due to the increase in the number of DNA molecules that were immobilized onto the SN matrix. On the other hand, the decrease in the signal following the 0.6 mg loading of SNs was due to additional amount of nanospheres without immobilized DNA has significantly reduced the electron transfer rate due to the non-conductive property of SNs [37].

The amount of glutaraldehyde linker used was based in glutaraldehyde portions where between 2% until 25% glutaraldehyde was used. Glutaraldehyde is a commonly used compound for protein and polymer modification by forming covalent bonds with amine functional groups to produce a more stable structure [38]. From Figure 4b, the biosensor response increased with the portion of glutaraldehyde from 2% to 10% as glutaraldehyde acts as a linker between DNA and SNs. The levelling off the DPV response at the portion of glutaraldehyde above 10% was attributed to the limited amount of DNA to connect with glutaraldehyde linker.

### 3.2. Optimization of DNA and MB Concentration

**Figure 5:**
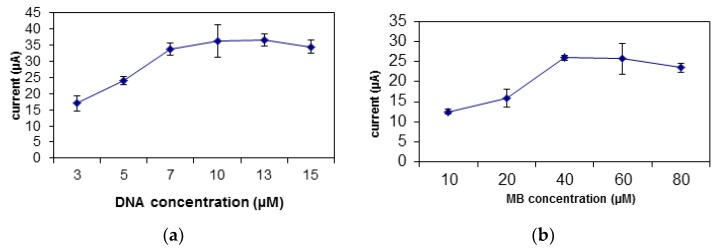
The optimization of the (**a**) DNA and (**b**) MB concentration to be used for the biosensor.

The increase in the concentration of DNA will increase the biosensor’s response due to the addition of binding spaces for the carcinogen and MB [33]. Figure 5a displays the rise in the biosensor’s response from DNA concentration of 3 µM to 10 µM. After this value, the biosensor’s response becomes constant due to the saturation of the binding spaces for DNA on the SNs [9]. There is a slight decrease in the biosensor’s response after the optimum DNA concentration of 10 µM. the biosensor’s response decreases after the optimum DNA concentration was achieved due to the excessive amount of unbound DNA that covers the surface of the electrode, and thus decreasing the amount of electron transfer [39]. 

MB plays an important role as redox indicator for the biosensor. There is an increase in the biosensor’s response from MB concentration of 10 µM to 40 µM, which can be related to MB capacity to bind to the DNA (Figure 5b). However, at MB concentration of 40 µM, all binding spaces on the DNA were filled and saturated with MB molecules, thus the biosensor’s response plateaus [40]. 

### 3.3. The Interaction Time of the MB and Carcinogen with Biosensor

**Figure 6:**
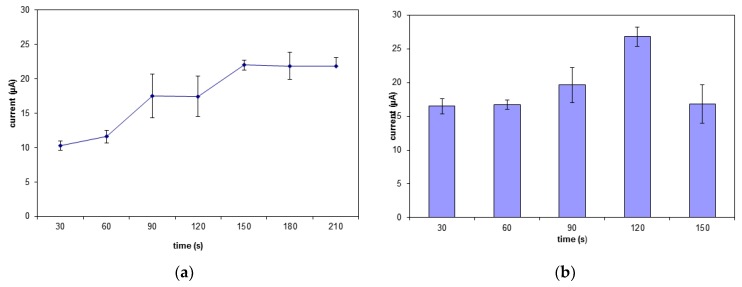
The interaction time of the (**a**) MB and (**b**) carcinogen with the biosensor.

The exposure time needed for MB to bind securely to the DNA was examined between 30 s to 210 s (Figure 6a). The biosensor’s response steadily increases from 30 s to 90 s before hitting a plateau. Thus, the accumulation time for MB molecules to occupy all the binding spaces on the DNA biomolecule was 90 s [41]. Figure 6b shows the time taken for the carcinogen to interact optimally with the DNA. The biosensor response gradually increased from 30 s to 120 s. This clearly shows that the amount of MB still exceeding the amount of carcinogen molecules immobilized onto the SNs. The biosensor response declines after 120 s due to the number of carcinogen molecules bound to DNA exceeding the number of MB molecules. The MB signal will decrease when the MB is unable to bind to the guanine mass and in this case due to the carcinogenic molecules saturated on the electrode surface [42,43].

### 3.4. Effect of pH, Ionic Strength, and Buffer Concentration

**Figure 7:**
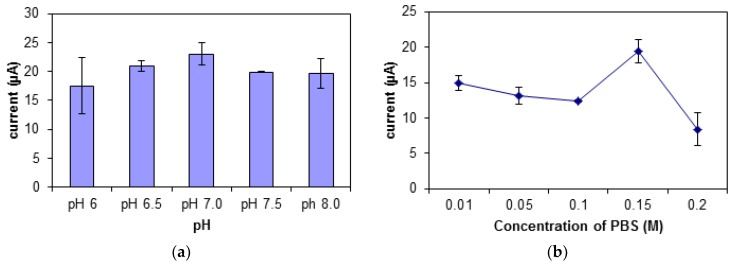

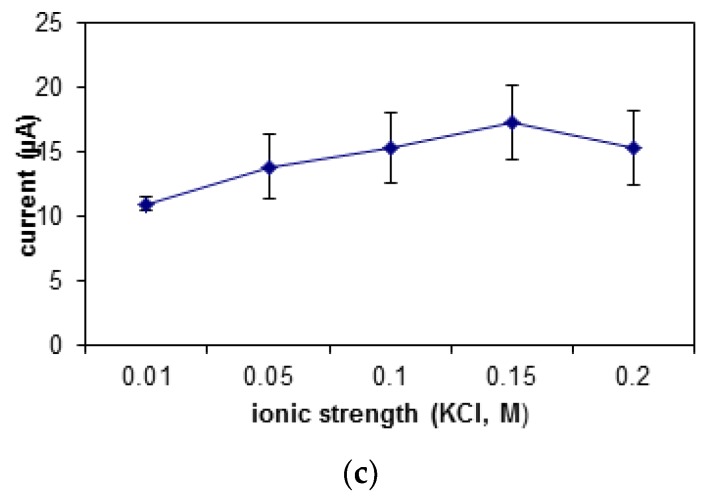
The effect of different (**a**) pH, (**b**) concentrations of PBS, and (**c**) KCl salt ionic strengths on the biosensor’s response.

Buffer solution that is acidic or alkaline is not suitable for the DNA biosensor. At a lower pH, the -phosphodiester bonds on the DNA will be deprotonated, thus decreasing the solubility of the DNA. This will cause difficulty in the formation of the DNA and MB complex, hence leading to a decrease in peak signal [44]. There is an increase in the biosensor’s response from pH 6.0 to pH 7.0 and a slight drop at pH 7.5 (Figure 7a). The increase in the biosensor’s response signifies an increase in the binding of MB molecules to the DNA. Thus, pH 7.0 was chosen as the optimum pH for this biosensor. 

The buffer used in this study is potassium phosphate buffer, PBS with potassium chloride, KCl as its ionic salt. There was an increase in the biosensor’s response shown in Figure 7b,c when the ionic strength and buffer concentration was increased from 0.01 M to 0.15 M. When the concentration of the buffer and its salt exceeds 0.2 M, the DNA may get curled, as there will be an interaction between the negatively charged backbones of the DNA with the counter ions in the buffer. This causes difficulty for the DNA, MB, and carcinogen to interact. However, at an optimum buffer concentration, the presence of cations will help to stabilize the shape of the DNA biomolecule [45]. At ionic salt concentration of 0.15 M, the DNA configuration was quite stable, hence giving easy access to the MB molecules and carcinogen to interact with the DNA.

### 3.5. Performance of the Biosensor on Cadmium Chloride (CdCl_2_)

**Figure 8:**
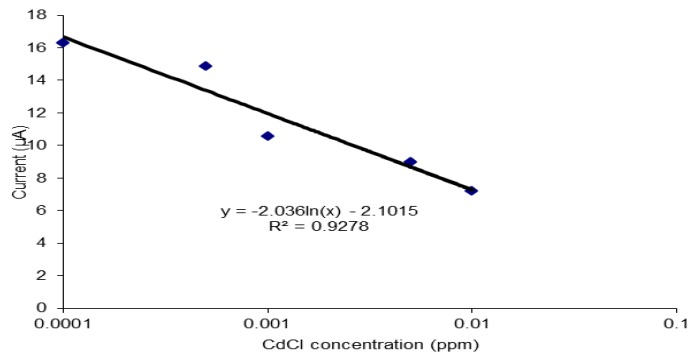
The performance of the biosensor on cadmium chloride (CdCl_2_).

The performance of the biosensor was tested on a heavy metal, cadmium (II) chloride (Figure 8). Cadmium was used in this study as it is a Class 1 carcinogen as classified by IARC. Since this is a confirmed carcinogen, it will have a strong affinity towards oligonucleotides. This is shown during the detection of cadmium (II) chloride using this biosensor where a wide dynamic range between 0.0005 ppm to 0.01 ppm was achieved with good linearity (R^2^ value of 0.959), sensitivity of 1.31 Δδ current/decade and detection limit of 0.0004 ppm. A DNA biosensor that could detect the presence of cadmium in the range of 1 × 10^−6^ ppm to 2 × 10^−5^ ppm and limit of detection of 3 × 10^−7^ ppm was built using ssDNA rich in adenine and thymine bases that was immobilized on the surface of Au electrode through Au-S covalent binding [46]. This proves that this biosensor gives a good response with carcinogens and is suitable for anti-tumor agent detection. 

### 3.6. Performance of DNA Biosensor with Synthesized Bioactive Compounds

**Figure 9:**
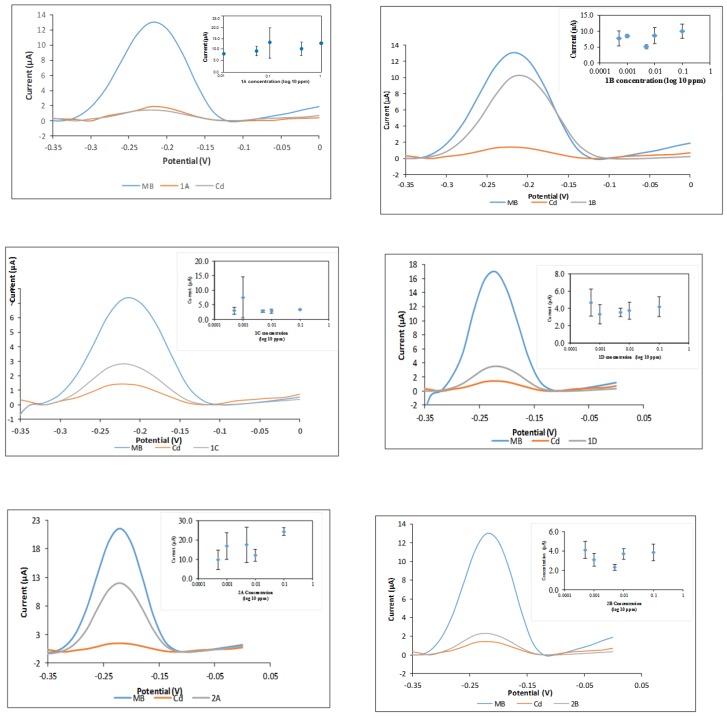
DNA biosensor with pyranopyrazole and quinoline compounds; ethyl 6-amino-5-cyano-4-phenyl-1,4-dihydropyrano[2,3-c]pyrazole-3-carboxylate (**1a**), ethyl6-amino-5-cyano-4-(4-ethylphenyl)-1,4-dihydropyrano[2,3-c]pyrazole-3-carboxylate (**1b**), ethyl6-amino-5-cyano-4-(furan-2-yl)-1,4-dihydropyrano[2,3-c]pyrazole-3-carboxylate (**1c**), ethyl6-amino-5-cyano-4-isopropyl-1,4-dihydropyrano[2,3-c]pyrazole-3-carboxylate (**1d**), 2-((7-chloroquinolin-4-yl)amino)ethanol (**2a**) and N-(2-bromoethyl)-7-chloroquinolin-4-amine (**2b**). * Large error bars show the non-carcinogenic compounds.

The performance of the DNA biosensor was tested with synthesized compounds as well as anti-tumor agents that were previously reported. The carcinogenicity analysis using a toxicity DNA biosensor is essential in order to verify whether these anti-tumor drugs are carcinogenic. Figure 9 shows a DPV peak at −0.22 V, which is the signal for the MB indicator in comparison to signals of the tested compounds and carcinogen heavy metal, cadmium chloride (CdCl_2_). In this system, the MB indicator will compete with carcinogen to bind with DNA. This means that if a substance is carcinogenic, the current signal or peak will be much lower than that of the MB indicator. Compounds **1a**–**1d** and **2a**–**2b,** which may be of biological importance, were used to investigate the performance of the DNA biosensor. In Figure 9, there is a clear decreasing peak in the presence of DNA, MB, and ethyl 6-amino-5-cyano-4-phenyl-1,4-dihydropyrano[2,3-c]pyrazole-3-carboxylate (**1a**). Similar results were obtained for compounds **1d** and **2b**. The current signals for these compounds are similar with the current signal for cadmium (carcinogen). The carcinogen molecules occupied the binding spaces of the DNA and reduced the amount of methylene blue molecules that can bind to the bases of the DNA [47]. These results have shown that **1a**, **1d**, and **2b** are classified as carcinogenic compounds. However, the current signal for 2-((7-chloroquinolin-4-yl)amino)ethanol (**2a**) is a little bit higher than methylene blue’s signal. The result shows that **2a** compound is classified as non-carcinogen compound. 

**Figure 10:**
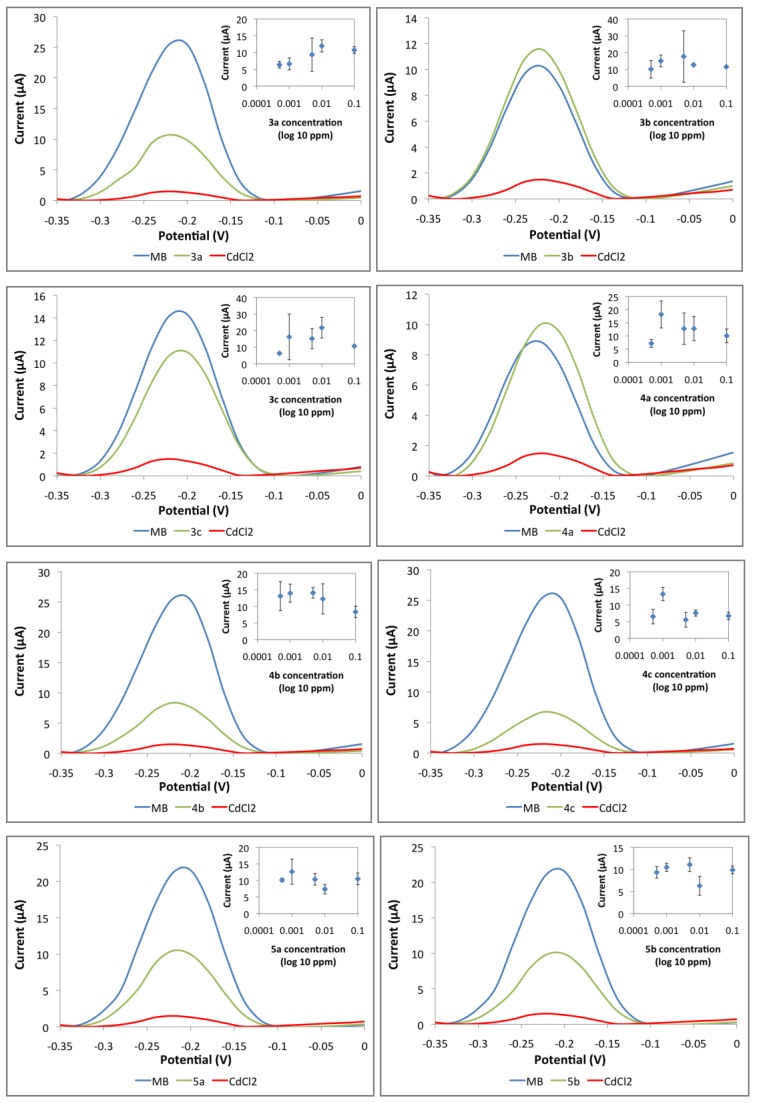
Performance of DNA biosensor with phthalides and bisindoles compounds; 3-[(1,3,3-Trimethylindolin-2-ylidene)methyl]isobenzofuran-1(3*H*)-one (**3a**), 3-[(5-Chloro-1,3,3-trimethylindolin-2-ylidene)methyl]isobenzofuran-1(3*H*)-one (**3b**), 6,7-Dimethoxy-3-[(5-chloro-1,3,3-trimethylindolin-2-ylidene)methyl]isobenzofuran-1(3*H*)-one (**3c**), 3-(1-Methylindol-3-yl)isobenzofuran-1(3*H*)-one (**4a**), 3-(5-Bromo-1-methylindol-3-yl)isobenzofuran-1(3*H*)-one (**4b**), 6,7-Dimethoxy-3-(5-bromo-1-methylindol-3-yl)isobenzofuran-1(3*H*)-one (**4c**), 2-(Bis(1-methylindol-3-yl)methyl)benzoic acid (**5a**) and2-(Bis(5-bromo-1-methylindol-3-yl)methyl)benzoic acid (**5b**). * Large error bars show the non-carcinogen compounds.

The current signal for compounds **3b** and **4a** are higher than the MB signal. The resulting current peak was produced because a substantial amount of MB molecules were able to be adsorbed onto the DNA phosphate backbone. Moreover, the tested compounds were not able to bind with DNA and hence, could not be detected by this biosensor. The initial findings suggest that **3b** and **4a** are non-carcinogenic compounds. On the other hand, the DPV peak for carcinogen sample CdCl_2_ showed a low current signal. The presence of carcinogens has filled up the binding sites on the DNA which consequently leave less space for the interaction between MB-DNA. All the other compounds tested showed a slightly lowered MB current signal. This initial finding suggests that compounds **3a**, **3c**, **4b**, **4c**, **5a**, and **5b** possess limited carcinogenic properties. Further biosensor performance tests were performed by screening the compounds with various concentrations to validate these initial findings. The insets in Figure 9 demonstrate the current signals of MB with different compound concentrations. Generally, the higher the carcinogen concentration, the lower the current signal. A decreasing trend will be shown on the graph of current signal against compound concentration (inset) if the compound is carcinogenic. However, none of the insets in Figure 10 display such a decreasing trend. In addition, the current peaks showed a relatively large deviations (error bars) considering that the noise has becoming more significant, considering the changes of the signal are very small as there is no binding with the toxicants, hence no competition binding with the MB. It is noteworthy that the response is more stable when there is a binding event. Those compounds indicating toxicity have lower errors compared with those with no toxicity. In the non-binding event, only the baseline signal is evaluated, which is less stable. The results of this investigation show that the DNA biosensor has successfully classified the compounds, into either carcinogenic or non-carcinogenic compounds. Relevant validation and interference studies for this biosensor have been conducted and published previously. This biosensor has been used for formaldehyde detection in mackerel fish extract and acrylamide determination in cassava chip extract. It has been compared with the standard Nash method, standard HPLC-UV method, and Ames test [47].

## 4. Conclusions

In this study, an electrochemical DNA biosensor with a 24 base guanine-rich nucleotide was constructed by using methylene blue as the indicator and silica nanoparticles as an immobilization platform. This toxicity electrochemical DNA biosensor was developed for the purpose of evaluating the carcinogenicity of anticancer compounds. The developed toxicity electrochemical DNA biosensor can detect carcinogens with the lowest limit of detection at 3 ppb. This biosensor can be a useful tool to monitor the toxicity of carcinogens as it can differentiate between carcinogenic and non-carcinogenic compounds. In future, the proposed toxicity electrochemical DNA biosensor could be used for screening anticancer compounds in biomedical fields.
